# Design and Fabrication of a Visible-Light-Compatible, Polymer-Based Photonic Crystal Resonator and Waveguide for Sensing Applications

**DOI:** 10.3390/mi9080410

**Published:** 2018-08-17

**Authors:** Jiayi Sun, Kenichi Maeno, Shoma Aki, Kenji Sueyoshi, Hideaki Hisamoto, Tatsuro Endo

**Affiliations:** 1 Department of Applied Chemistry, Osaka Prefecture University, Osaka 599-8531, Japan; swb02080@edu.osakafu-u.ac.jp (J.S.); su108052@edu.osakafu-u.ac.jp (K.M.); akiel0719@yahoo.co.jp (S.A.); sueyoshi@chem.osakafu-u.ac.jp (K.S.); hisamoto@chem.osakafu-u.ac.jp (H.H.); 2 Japan Science and Technology Agency (JST) Precursory Research for Embryonic Science and Technology (PRESTO), Tokyo 102-8666, Japan

**Keywords:** photonic crystal, resonator, waveguide, layer-by-layer method, optical sensor

## Abstract

In this paper, we have proposed a polymer-based photonic crystal (PhC) resonator, with multiple sizes of cavities, and a waveguide to be used as highly sensitive optical sensor components. Properties of the proposed PhC were simulated by the finite-difference time-domain method, and the polymer-based PhC resonator and waveguide were fabricated on a photoresist (polymer) by electron beam lithography, which was prepared on an Au-layer-deposited Si substrate. We detected the resonant light that penetrated through the waveguide and was trapped in the PhC resonator. Optical characteristics of the fabricated PhC were evaluated by detecting the polymer layer deposition process by using the layer-by-layer (LbL) method to deposit polymer layers. As a result, by using an optimized design of a polymer-based PhC resonator with a long cavity (equivalent to a defect of three holes), the PhC structure changes caused by LbL deposition lead to changes in resonant light wavelength (peak shift: 5.26 nm/layer). Therefore, we suggest that a PhC resonator and a waveguide is applicable as an optical sensor.

## 1. Introduction

Photonic crystal (PhC) is a periodically dielectric nanostructure that has attracted significant interest recently because of its unique optical properties. Light of specific wavelengths cannot penetrate through a PhC because of the Bragg reflector structure of the PhC. This limited range of wavelength has been called the photonic band gap (PBG) since Yablonovitch coined the term in 1987 [1,2]. By controlling the penetration of light using the PBG, the PhC is applied to optical elements, such as filters [3] and splitters [4]. Furthermore, the PBG is sensitive and can be affected by surrounding refractive index changes, which lead the PhC to be able to function as a highly sensitive optical sensor [5,6,7]. In addition, by introducing a line defect into the PhC, a PhC waveguide can be formed, and the light in the PBG range can penetrate through the waveguide. Hence, the PhC waveguide has been used for sensing applications, such as gas sensors [8,9] and biosensors [10,11]. Moreover, a PhC resonator realized by introducing point defects into the PhC can contribute to the trapping and amplification of light within the PBG range. Based on these features, the PhC resonator is reported to function in various fields, such as nanolaser development [12] and quantum information processing [13].

Conversely, the substrate usually used to fabricate a PhC, such as Si [14] and GaInAsP [15], prevents the PhC from widespread applications. For example, to fabricate a Si-based PhC, the widely utilized methods for drawing patterns are electron beam lithography (EBL) and reactive ion etching (RIE). However, these methods require highly costly apparatus and sophisticated fabrication processes. Furthermore, a Si-based PhC limits the optical detection wavelength to the infrared range [14]. Therefore, we propose to use a polymer substrate instead of common Si or GaInAsP to fabricate a PhC, which avoids the use of highly costly apparatus, allows for simple fabrication using nanoimprint lithography (NIL), and also allows for optical detection in the visible wavelength range [16,17,18,19,20,21,22,23].

Compared with semiconductor-based PhC sensors, polymer-based PhC sensors can be fabricated cost effectively and easily by the NIL process. Additionally, the optical characterization of the sensor can be performed using a simplified optical setup, such as a handy-type spectrometer or smartphones.

In this paper, we propose the design and fabrication of a polymer-based PhC resonator and waveguide for optical sensor applications. We have carried out some preliminary experiments and found that distinguishing between the light that penetrated through the waveguide and the ambient light was difficult due to the low refractive index of the polymer [24]. However, fabrication of the resonator structure nearby the PhC waveguide is expected to allow the penetrated light to be localized at the resonator, which makes optical detection more accurate and easier. Here, we simulated a PhC property by the three-dimensional, finite-difference time-domain (FDTD) method first, then, according to the results of the simulations, a polymer-based PhC resonator and waveguide were fabricated by using an electron beam resist as a model of the polymer material. By detecting the trapped light at the resonator, we confirmed the usability of the polymer material, which suggests the possibility of using many other functional polymers as waveguide-based PhC sensor materials. Chemical sensing characteristics were evaluated by detecting the polymer layer deposition process using the layer-by-layer (LbL) method [25].

## 2. Materials and Methods

### 2.1. Materials

For fabrication of the polymer-based PhC resonator and waveguide, sulfuric acid (95% (*v*/*v*)), hydrogen peroxide (30% (*v*/*v*)), acetone (99.7% (*v*/*v*)), 2-propanol (99.9% (*v*/*v*)), and xylene were purchased from Wako Pure Chemicals (Osaka, Japan). ZEP520A as an electron beam resist was purchased from ZEON Corp. (Tokyo, Japan). For applying the LbL method, poly allylamine hydrochloride (PAH) and poly sodium 4-styrene sulfonate (PSS) were purchased from Sigma-Aldrich (Tokyo, Japan).

### 2.2. Apparatus

For fabricating the PhC resonator and waveguide, an ELS-7500 from Elionix Inc. (Tokyo, Japan) was used. For observing the PhC resonator and waveguide fabricated by EBL, a Field emission scanning electron microscope (FE-SEM) (JSM-7610F, JEOL Ltd., Tokyo, Japan) was used.

### 2.3. Method

#### 2.3.1. Simulation Analysis

**Figure 1:**
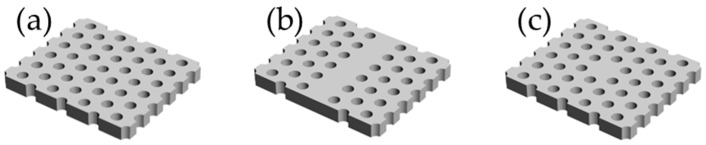
Basic design of photonic crystal (PhC) devices for simulation. (**a**) PhC with an air hole array, (**b**) PhC waveguide, (**c**) PhC resonator.

Before fabricating the polymer-based PhC resonator and waveguide, a simulation of the PhC was conducted with the FDTD (Lumerical Solutions Inc., Vancouver, BC, Canada) method to confirm the wavelength range of the PBG. The basic structure of the PhC used in this work is shown in Figure 1a. First, to confirm the penetration of light within the PBG wavelength range, we designed a PhC waveguide, shown in Figure 1b, by introducing a line defect from a normal PhC structure. The refractive index (*n*) of the air hole was set to 1.0, while the refractive index of the PhC base material was 1.5, which is close to that of the photo resist, ZEP520A, used in this work. The radius of the air hole (*r*), the lattice constant (*a*), and the thickness (*h*) were set at 84 nm, 300 nm, and 200 nm, respectively. The wavelength of the irradiated light was set in the visible range (400–700 nm), and the electric field intensity was obtained by setting the monitor at the exit of the waveguide. After confirming the visible light penetration, we simulated the localization and resonance of the visible light at the resonator. The basic design of the PhC resonator is shown in Figure 1c. The light source was set at the same position, but the position of the monitor was at the cavity this time, which simulated the electric field intensity.

Considering the impracticality of PhC fabrication without a substrate as in the simulation demonstrated above, we simulated the effect of four different substrates Si, Au, Ag, Cu, and compared the electric field intensities [26].

To enhance the efficiency of light confinement at the resonator, a number of point defects was simulated. By varying the number of point defects from 1 to 5 in the simulation, we investigated the influences on the electric field intensity at the PhC resonator.

#### 2.3.2. Fabrication of PhC Using Electron Beam Lithography

Utilizing the optimized design, actual fabrication was conducted by EBL. Before exposure to the electron beam, the substrate was first cleaned by the piranha solution (a mixture of sulfuric acid and hydrogen peroxide) for 30 min at 150 °C; subsequently, ultrasonic cleaning in acetone was conducted in a clean room. After washing by ultra-pure water and 2-propanol, ZEP520A was coated onto the substrate by spin coating (1000 rpm, 120 s), and then exposed to an electron beam. In the actual fabrication process, we combined the waveguide and the resonator pattern together as introduced above. The distance between the waveguide and resonator was set to 11 periods of nanopattern so that light can be confined most efficiently at the resonator without being influenced by the light penetrating through the immediately adjacent waveguide. The exposed region was removed by xylene, and an air hole array structure was fabricated. Confirmation of the fabricated structure and measurement of *r* and *a* were carried out by FE-SEM.

#### 2.3.3. Evaluation of PhC Resonator and Waveguide

**Figure 2:**
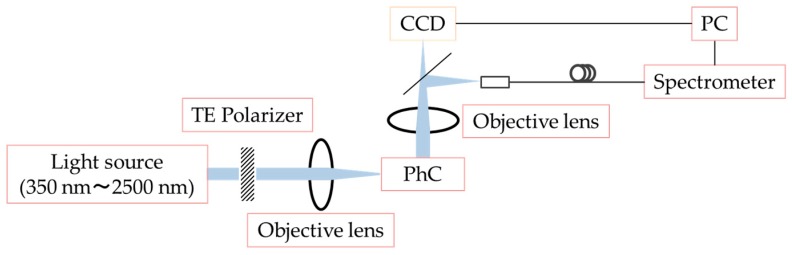
Optical setup used to detect resonant light. CCD, charge-coupled device; PC, personal computer, TE, Transverse Electric.

Figure 2 shows the optical setup used to conduct the detection of resonant light in this work. Incidental white light was irradiated from the horizontal direction and detected by a charge-coupled device (CCD) camera (Hitachi KP-D20BP, Edmund Optics Inc., Barrington, NJ, USA) and the spectrometer (CS100/M, Thorlabs Inc., Newton, NJ, USA) described above.

To evaluate the performance of the PhC resonator and waveguide as a sensor, the LbL method was utilized. As the LbL method was performed, a multilayer was formed by the polymer monolayers, and at the same time, a decrease in the hole size of the PhC occurred. Here, we used the PAA aqueous solution (3 mg/mL) as the polycation solution, and the PSS aqueous solution (3 mg/mL) as the polyanion solution, and alternately introduced one drop on the surface of the PhC resonator waveguide device. The resonant light was measured at each step of layer formation.

## 3. Results

### 3.1. Simulation

**Figure 3:**
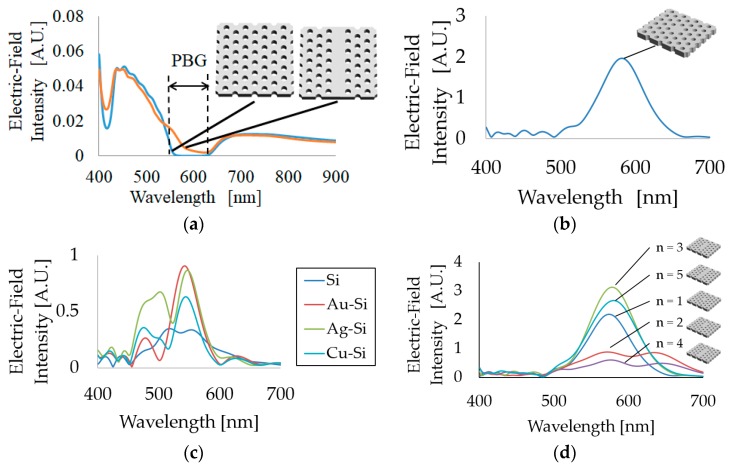
Results of simulations conducted to optimize PhC structure. (**a**) PhC and PhC waveguide, (**b**) PhC resonator, (**c**) PhC resonators with different substrates, (**d**) PhC resonators with different numbers of cavities. PBG, photonic band gap.

Using the FDTD method, electric field intensities obtained by the PhC structure with and without the waveguide were simulated, and the results are shown in Figure 3a. From Figure 3a, the wavelength range at 560–640 nm showed almost zero electric field intensity, suggesting that this range corresponds to the PBG range. On the other hand, the PhC with a waveguide shows that within the PBG range, light penetrated slightly through the waveguide. Therefore, when a polymer-based PhC structure is designed using the proposed conditions (*a* = 300 nm, *r* = 84 nm, *h* = 200 nm), visible light penetrates and is transmitted through the waveguide.

An identical structure to this PhC waveguide without a line defect but with a point defect was also built by FDTD simulation and simulated as a PhC resonator. The results of this simulation are shown in Figure 3b, and they demonstrate that the maximum wavelength of resonant light and the full width at half maximum (FWHM) are 590 nm and 68 nm, respectively. This resonant wavelength range exactly covers the light that penetrated through the waveguide. Therefore, the PhC resonator waveguide with the structure described above is expected to penetrate and localize visible light in actual experiments.

Although visible light penetration was confirmed by simulation, polymer-based PhC fabrication without a substrate is unrealistic due to the soft nature of the polymer material. Hence, it is essential to use a substrate under a polymer-based PhC. When the substrate has a refractive index higher than that of the PhC material, where the refractive index is approximately 1.5 in this work, penetrated light tends to leak to the substrate from the PhC. On the other hand, if a substrate with a low refractive index in the visible light range, such as a metal substrate, is used for a polymer-based PhC, light would be reflected back from the metal substrate and total reflection would occur in the polymer layer. As a result, light is confined with high efficiency in the PhC and expected to be detected with higher intensity and a narrow FWHM. Therefore, four different substrates with different refractive indexes (Si: 3.94, Au: 0.21, Ag: 0.13, and Cu: 0.41) at 600 nm were utilized to simulate the light confinement ability of the PhC resonator; the thickness of the metal on Si was set at 200 nm, except for the Si substrate. In this case, Figure 3c shows the results of each substrate, and shows that the metal substrate exhibits a significantly better confinement ability than a Si substrate. Moreover, compared to Ag and Cu, Au shows a stronger intensity, although the refractive index is higher than that of Ag, which is due to the extinction coefficient of Ag—approximately 4.0—which is significantly larger than that of Au—approximately 0.05—at 600 nm. Based on this result, we decided to use Au-layer-deposited Si as a substrate of the PhC for the actual fabrication and measurement process.

Figure 3d shows the results of the resonant intensity of the PhC resonator where the number of point defects is varied from 1 to 5. From the previous study [26], the distance between the resonator and the waveguide will be affected by the coupling and confinement efficiency. In this study, the optimized distance (11*a* (3.3 μm)) (*a*: Lattice constant) was applied for the fabricated design of the PhC resonator and waveguide. When an even number of point defects was introduced, light waves overlapped so that the light confined in the PhC shows destructive interference, making the spectra exhibit low intensity and a wide FWHM. Conversely, when an odd number of point defects was introduced, especially three defects, constructive interference was demonstrated by the waves, resulting in stronger intensity and a narrower FWHM. Based on this result, we chose a three-defect PhC resonator for sensor applications.

### 3.2. Fabrication of PhC

**Figure 4:**
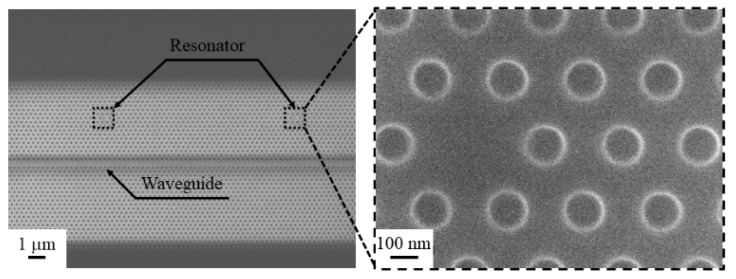
SEM image of polymer-based PhC resonator and waveguide.

Based on the simulated results, we carried out fabrication of the PhC resonator and waveguide using a photo-resist polymer. For optimizing the EBL condition, a Si substrate was used for confirming the pattern drawn by EBL, and we found that the designed hole radius of *r* = 80 nm and the exposure time of dose = 0.75 μs/dot were the most appropriate conditions, which resulted in *r* = 82.5 nm as confirmed by FE-SEM. However, under the same fabrication conditions, the hole radius was increased to 100 nm when the Au–Si substrate was used. This change of size may be caused by the emission of free electrons from Au after accepting the external energy applied by EBL. In spite of the larger radius, we still confirmed the waveguide’s, resonator’s, and PhC’s structure from the FE-SEM image (Figure 4) clearly, therefore confirming successful fabrication of the PhC resonator and waveguide.

### 3.3. Microscopic Observation and Measurements

**Figure 5:**
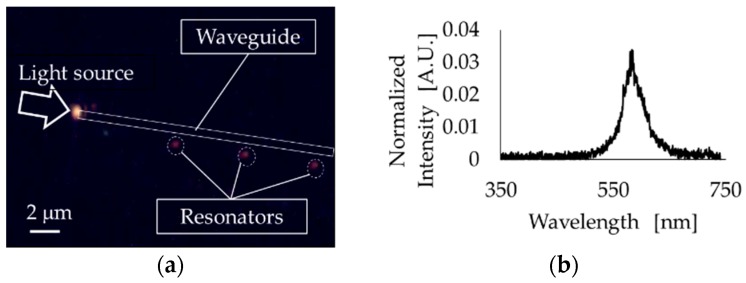
Microscopic observation of the PhC resonator and waveguide (**a**) and spectrum of the detected light at resonator (**b**).

Using the fabricated PhC resonator and waveguide, the resonant light at the resonator was observed and detected by the setup shown in Figure 2. Figure 5a shows the dark-field image, and the resonant light was clearly observed at the resonator position. Figure 5b shows the spectrum of resonant light, from which resonated light with λ = 584 nm and FWHM = 48 nm was confirmed.

**Figure 6:**
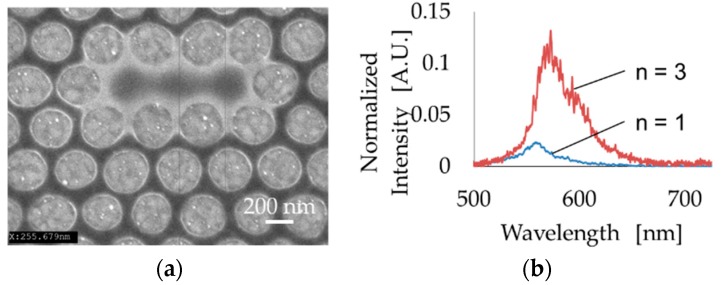
(**a**) Fabrication of the three-defect PhC resonator and waveguide. (**b**) Comparison of intensity with one and three defects.

**Figure 7:**
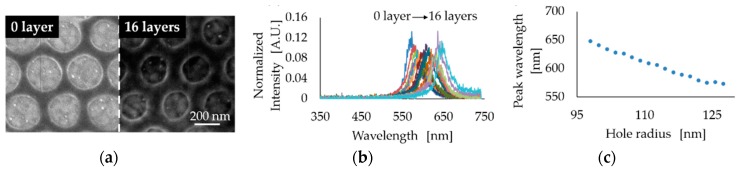
Evaluation of the three-defect, polymer-based PhC resonator and waveguide by the layer-by-layer (LbL) method. (**a**) FE-SEM images before and after LbL deposition, (**b**) Resonant spectral changes during the LbL process, (**c**) Relationship between PhC hole size and resonant wavelength.

After confirming the optical characteristics of the one-defect PhC resonator and waveguide, we fabricated a three-defect PhC device since we expected it would exhibit precise localization of light based on the simulated results shown in Figure 3d. Figure 6a shows the three-defect PhC device we fabricated. The resonant light spectrum was compared to that of the one-defect device. Figure 6b shows the better capability of light localization in the three-defect PhC. Therefore, a LbL deposition experiment was carried out using the three-defect PhC resonator and waveguide. Here, we repeated the dropping of polycation and polyanion solutions 16 times. PhC hole size and FE-SEM images before and after the LbL process are shown in Figure 7a. During this process, the hole radius changed from 128 nm to 98 nm, and at the same time, the resonant light spectrum also changed (Figure 7b). The relationship between the hole radius and resonant light wavelength is shown in Figure 7c. Redshift of resonant light corresponding to the change of the hole radius was observed. The changing rate of the resonant wavelength was calculated to be 1.4 nm shift for 1 nm decrease of hole radius diameter. These results demonstrate that the proposed device can detect the absorption of a few nm of target molecules, such as DNAs and proteins. Hence, in the future, using this device, the development of a highly sensitive optical sensor can be expected.

In this study, by using an LbL deposition experiment, approximately 4.9 nm of PAH/PSS multilayers were deposited in the hole by SEM imaging. From the previous report using similar experimental conditions [27], similar-thickness PAH/PSS multilayers were deposited. In addition, to compare the experimental result of peak wavelength shift due to the deposition of PAH/PSS multilayers, a simulation analysis was carried out. As a result, by the deposition of PAH/PSS multilayers, a similar peak shift could be observed (data not shown). Furthermore, deposition of the PAH/PSS multilayers on the top of the PhC resonator and waveguide also attributed to the red shift of the peak wavelength. When the PAH/PSS multilayers were deposited, a decrement in the hole radius and an increment in the PhC slab thickness occurred simultaneously. Hence, the refractive index per unit volume will be increased. This increment of refractive index per unit volume and size change induces the PBG shift as a peak wavelength shift.

From these characteristics of PhC resonator and waveguide, for sensing applications, monitoring of the peak shift due to the surrounding refractive index seems to be suitable. From the previous reports, Quan et al. [28,29,30] and Baba et al. [31] had successfully developed a super-sensitive biosensor (single molecular level). However, to perform highly sensitive detection at the single molecular level, the peak sharpness will be affected by the sensitivity. To realize the detection of a single molecule of a target using this PhC resonator and waveguide, more detailed investigations, such as on device design, base materials, and fabrication conditions, are required.

## 4. Conclusions

In this work, a visible-light-compatible and polymer-based PhC resonator and waveguide was designed and fabricated successfully by EBL. To enhance the efficiency of light localization, an Au-deposited Si substrate was used, and the optimal defect number was determined to be 3. By detecting light at the resonator position, visible light resonance was observed and measured successfully. Furthermore, basic optical characteristics of the polymer-based, three-defect PhC resonator and waveguide were evaluated by conducting LbL deposition. We confirmed that PhC hole size changes contribute to resonant light shift, which suggests the possibility of using the PhC resonator and waveguide as an optical sensor.

Based on these experimental results, fabrication of the PhC resonator and waveguide was done by applying NIL, which is cost-effective and has high reproducibility. In the future, the NIL-based PhC resonator and waveguide will be applicable to simple and disposable optical sensors for medical applications using an antigen–antibody reaction and DNA hybridization.

In addition, based on the detection principle of a PhC resonator and waveguide-based optical sensor, several applications, such as environmental monitoring [32] and food control [33], can be performed to extend the PhC resonator and waveguide’s use beyond medical applications. For these applications, volatile organic compounds (VOCs) and toxic substances can be detected by using the PhC resonator and waveguide.
